# How many fluorophores are required to achieve AIE?

**DOI:** 10.1039/d5sc04053a

**Published:** 2025-10-21

**Authors:** Tangxin Xiao, Zehuan Huang, Liangliang Zhang, Shuang Jin, Xiaoyi Chen, Hongwei Qian, Guanglu Wu, Jade A. McCune, Oren A. Scherman

**Affiliations:** a Melville Laboratory for Polymer Synthesis, Yusuf Hamied Department of Chemistry, University of Cambridge Cambridge CB2 1EW UK oas23@cam.ac.uk; b Jiangsu Key Laboratory of Advanced Catalytic Materials and Technology, School of Petrochemical Engineering, Changzhou University Changzhou 213164 P. R. China; c State Key Laboratory of Supramolecular Structure and Materials, College of Chemistry, Jilin University Changchun 130012 P. R. China

## Abstract

Aggregation-induced emission (AIE) of organic fluorophores is of broad interest for research in areas including chemistry, biology and materials science. Previous reports have mainly focused on enhancing fluorescence of various AIE entities including small molecules, biomacromolecules and nanoparticles. However, there remains a fundamental lack of understanding of the initial aggregation steps AIE cores undergo. Herein, we report the modification of an archetypal AIE fluorophore (tetraphenylethylene, TPE) with aryl pyridinium salts to form a TPE derivative. Employing cucurbit[*n*]uril macrocyclic hosts in the presence of this TPE derivative enables the controlled formation of discrete monomeric and dimeric structures, shedding light onto the early stages of aggregation. The discrete TPE dimer exhibits unprecedented photophysical behaviour with strong fluorescence in both the solid and liquid states. This supramolecular clamping strategy results in through-space dimerisation enhanced emission, providing detailed insights applicable for the design and formation of functional fluorescent (bio)materials.

## Abstract 2

Aggregation-induced emission (AIE) of organic fluorophores is of broad interest for research in areas including chemistry, biology and materials science.

## Graphical abstract

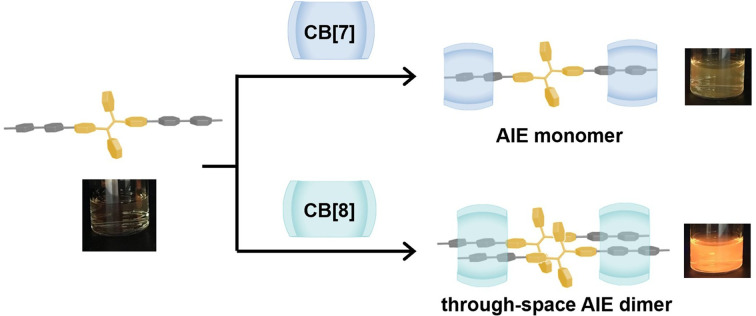

Over the past decade, aggregation-induced emission (AIE) containing materials^1^ have attracted attention on account of their promising applications in bioimaging,^2^ photodynamic therapy,^3–5^ light harvesting,^6–8^ and optoelectronic devices.^9^ Classes of AIE molecules that incorporate a propeller-like molecular conformation have been commonly employed including cyanostilbene,^10,11^ pentaphenylsilole^12^ and tetraphenylethylene (TPE, Fig. 1a).^13^ Suppression of the conformational rotation of these propeller structures, referred to as restriction of intramolecular motion (RIM), is critical to prevent non-radiative deactivation pathways of AIE molecules in the excited state, responsible for the AIE phenomenon.^14,15^ Hence, AIE molecules show intense emission in their aggregated state including as solvent dispersed nanoparticles,^16,17^ films,^18^ gels,^19^ and crystalline materials.^20^ To further enhance the AIE effect, researchers have immobilised the propeller conformation through-bond into both infinite networks (*e.g.* supramolecular polymers,^21^ metal organic frameworks,^22^ covalent organic frameworks^23^ and supramolecular organic frameworks^24^) and discrete entities (*e.g.* covalently bonded macrocycles^25,26^ and coordination cages^27–31^). Although numerous strategies, both covalent and noncovalent, have been shown to activate AIE at the molecular or supramolecular level, the minimum number of molecules required to induce luminescence *via* through-space aggregation remains unexplored (Fig. 1b).

**Fig. 1:**
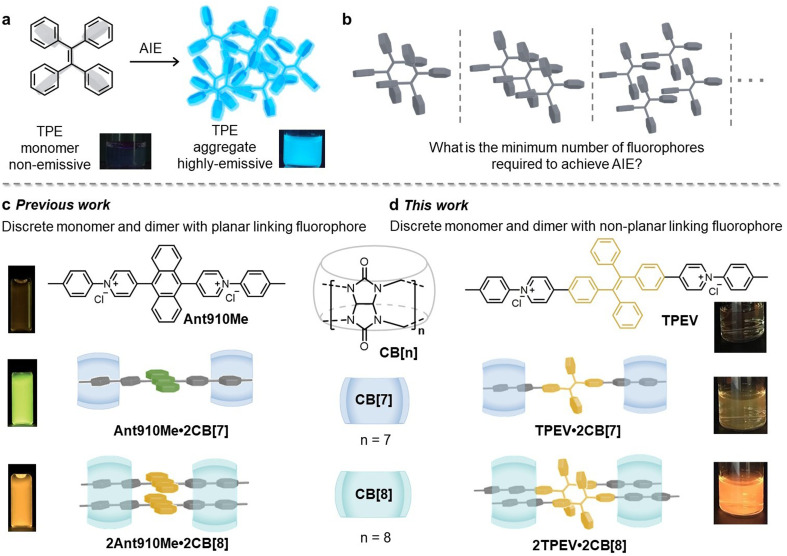
(a) Chemical structure of TPE and its AIE behaviour evidenced by two photographs of TPE, non-emissive in THF and strongly emissive in THF/H_2_O (v/v = 1/9), respectively. (b) Schematic of the early stages of TPE aggregation. (c) Schematic representation of Ant910Me and its host–guest complexes with CB[7] and CB[8], and their fluorescent images. (d) Schematic representation of TPEV and its host–guest complexes with CB[7] and CB[8], and their fluorescent images.

Supramolecular macrocycles have been used to regulate the luminescence of organic chromophores.^32–36^ Among these macrocycles, cucurbit[*n*]urils (CB[*n*]) have attracted particular attention on account of their high binding affinities with a wide range of guest moieties in aqueous media.^37–40^ The smaller CB[*n*] homologues (*n* = 6–7) can encapsulate one guest molecule within their hydrophobic cavity, however, the larger CB[8] homologue is capable of simultaneous encapsulation of two guest moieties within its cavity to form ternary complexes.^41–45^ Based on this, assorted CB[*n*]-based photoluminescent materials have been reported.^46–55^ Planar fluorophores typically undergo π–π stacking in concentrated solution or in the solid state, exhibiting an aggregation-caused quenching (ACQ) effect. By employing CB[*n*] as supramolecular hosts to sequester fluorophore-containing guests, we have previously constructed highly emissive discrete monomeric and dimeric complexes, particularly when using structurally rigid guests with planar fluorophore cores (Fig. 1c).^56–59^ These host–guest systems (*e.g.* Ant910Me·CB[*n*]) generally exhibit the following characteristics: (1) CB[7] forms a 2 : 1 complex with the guest, resulting in discrete monomeric species with strong emission; (2) CB[8] forms a 2 : 2 complex with the guest, leading to discrete dimeric species with strong bathochromic emission. Leveraging these 1 : 2 and 2 : 2 binding modes, we propose the formation of discrete monomer and dimer species with a non-planar linking fluorophore to study the initial stages of through-space AIE aggregation (Fig. 1d).

## Results and discussion

**Fig. 2:**
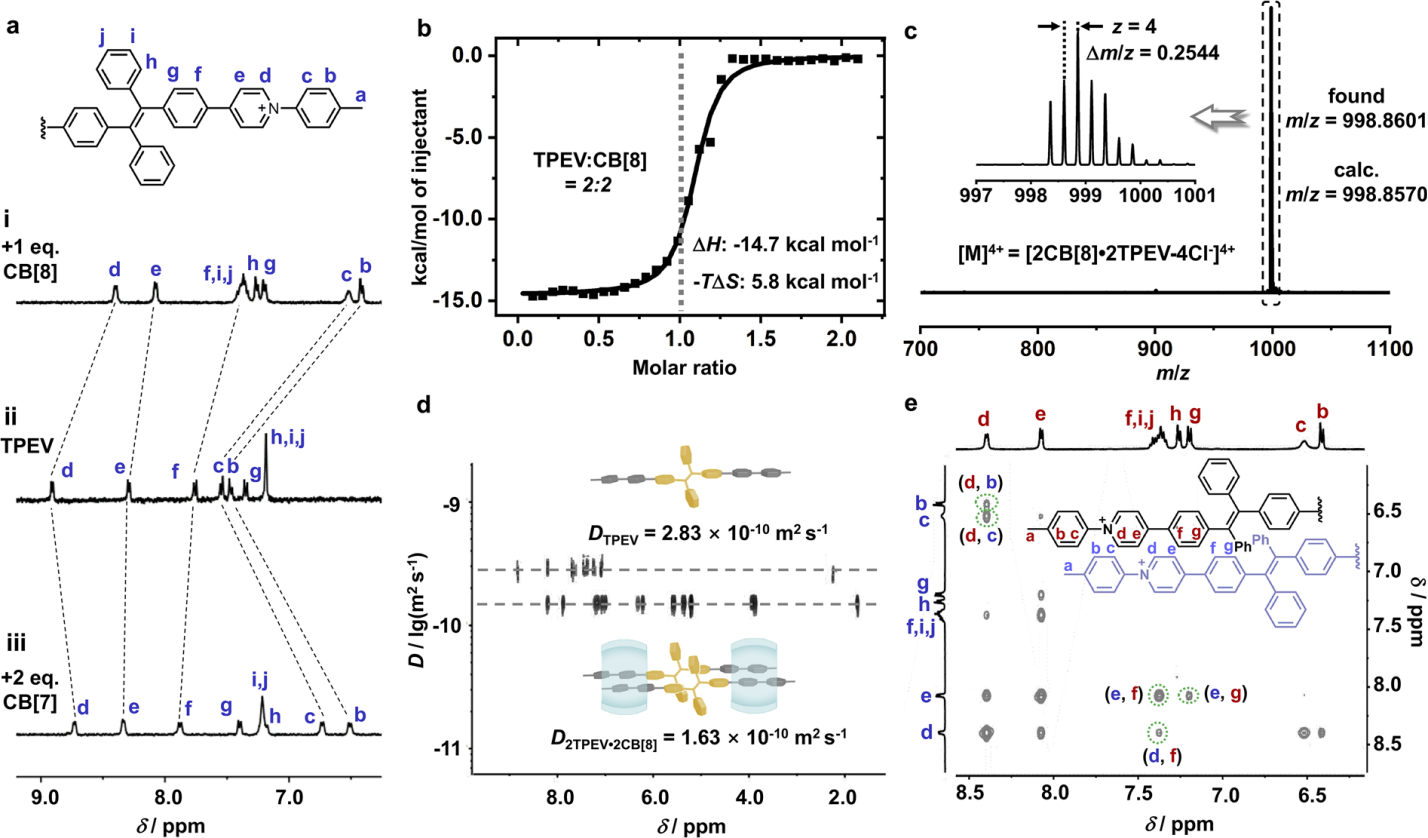
(a) Partial chemical structure of TPEV and ^1^H NMR spectra (400 MHz, 298 K, D_2_O) of (i) a TPEV complex with 1 eq. of CB[8], (ii) free TPEV, and (iii) TPEV with 2 eq. of CB[7] ([TPEV] = 200 μM). (b) ITC plot and fitted curve (H_2_O, 298 K) obtained through titration of TPEV (0.50 mM) into CB[8] (0.05 mM). (c) HR-ESI-MS of 2TPEV·2CB[8] showing an ion peak with four charges (*z* = 4) and a matched *m*/*z* centred at 998.8601, which confirms the formation of [2TPEV·2CB[8]-4Cl^−^]^4+^. (d) DOSY spectra (400 MHz, 298 K, D_2_O) of TPEV and 2TPEV·2CB[8] (e) Partial 2D NOESY spectrum (500 MHz, 298 K, D_2_O) of 2TPEV·2CB[8].

The TPE chromophore was selected as the AIE core to study on account of its excellent photophysical properties as well as the ease of synthetic modification.^60^ To enable the formation of a TPE monomer through 1 : 2 CB[7] complexation and a TPE dimer through 2 : 2 CB[8] complexation,^56,57,59^ a ditopic guest molecule TPEV containing a TPE fluorophore core flanked by two arylpyridinium groups was designed and synthesised. In contrast to previously reported 2 : 2 complexes with planar fluorophore cores, TPE is a 3D-shaped, bulky molecule with a propeller conformation that might prevent the formation of a 2 : 2 complex. To investigate this question, NMR spectroscopy was employed to identify the relative position of the guest moieties within the CB[8] cavity in the complex form. When 1 eq. CB[8] was added to an aqueous solution of TPEV, the protons from the tolyl and pyridinium moieties exhibited significant upfield shifts (Fig. 2a), and the protons (H_x_ and H_y_) on the portal of CB[8] split into two sets of peaks, suggesting the formation of a host–guest complex with tolyl and pyridinium moieties located inside the CB[8] cavity. Therefore, ^1^H NMR preliminarily confirmed the 2 : 2 complexation between TPEV and CB[8] (Fig. S7 and Table S1).

Isothermal titration calorimetry (ITC) further showed the 1 : 1 stoichiometry of TPEV and CB[8] (Fig. 2b and S9). A relatively large enthalpy change (Δ*H* = −14.7 kcal mol^−1^) was obtained in the case of 2TPEV·2CB[8] complexation, consistent with the reported Δ*H* values of typical 2 : 2 complexes.^59^ High-Resolution Electrospray Ionisation Mass Spectrometry (HR-ESI-MS) spectrum of 2TPEV·2CB[8] showed a set of ion peaks with four positive charges (*z* = 4) with a *m*/*z* value centred at 998.8601 (Fig. 2c), consistent with the calculated *m*/*z* for [2TPEV·2CB[8]-4Cl^−^]^4+^. Similar to the binding mode of Ant910Me·2CB[7], complexation of TPEV and CB[7] in a 1 : 2 mode was first confirmed by ^1^H NMR (Fig. 2a, S13 and Table S1). Titration of TPEV into CB[7] showed a clear transition at a 1 : 2 molar ratio (Fig. S15), indicative of the formation of a 1 : 2 complex. The structure was also confirmed by HR-ESI-MS (Fig. S14). All these results indicate that TPEV·2CB[7] is a discrete monomeric form of TPE, representing an ideal reference for the TPE dimer in 2TPEV·2CB[8] to study the “first step” in through-space aggregation of TPE.

Diffusion ordered ^1^H NMR spectroscopy (DOSY) experiments were further performed. The larger complex usually possesses the smaller diffusion coefficient. DOSY spectrum of TPEV (Fig. S6), TPEV·2CB[7] (Fig. S18) and 2TPEV·2CB[8] (Fig. 2d and S12) all exhibit only one set of well-ordered signals, respectively, indicating that only a single species is exclusively formed in each case. The diffusion coefficient values (*D*) of unbound guest (TPEV), 1 : 2 binding complex (TPEV·2CB[7]), and 2 : 2 binding complex (2TPEV·2CB[8]) in aqueous solution are calculated to be 2.83 × 10^−10^ m^2^ s^−1^, 1.87 × 10^−10^ m^2^ s^−1^ and 1.63 × 10^−10^ m^2^ s^−1^, respectively. This decreasing trend is fully consistent with their increase in size, which further supports that the resultant 2TPEV·2CB[8] complex involves of two TPEVs held together by two CB[8] hosts.

Proximal stacking conformation of two TPEV monomers within the 2 : 2 complex is further evidenced by off-diagonal correlations in NOESY. In the NOESY spectra of TPEV (Fig. S5) and TPEV·2CB[7] (Fig. S17), only the correlations between neighbouring protons could be observed, such as H_b_–H_c_, H_d_–H_e_, H_e_–H_f_, and H_f_–H_g_. Compared to TPEV and TPEV·2CB[7], the NOESY spectrum of 2TPEV·2CB[8] displayed additional correlation information (Fig. 2e and S11). Several sets of NOE signals between tolyl and pyridinium moieties (H_b_–H_d_ and H_c_–H_d_), as well as pyridinium and phenylene moieties (H_d_–H_f_, H_e_–H_f_ and H_e_–H_g_) appeared. This indicates the proximity of the tolyl group on one of the TPEV molecules within the 2 : 2 complex to the pyridinium moiety of the other TPEV molecule. This finding further elucidates that, despite the 3D propeller structure of the TPE group, CB[8] can still facilitate the aggregation of two TPEs through space in a 2 : 2 pattern.

**Fig. 3:**
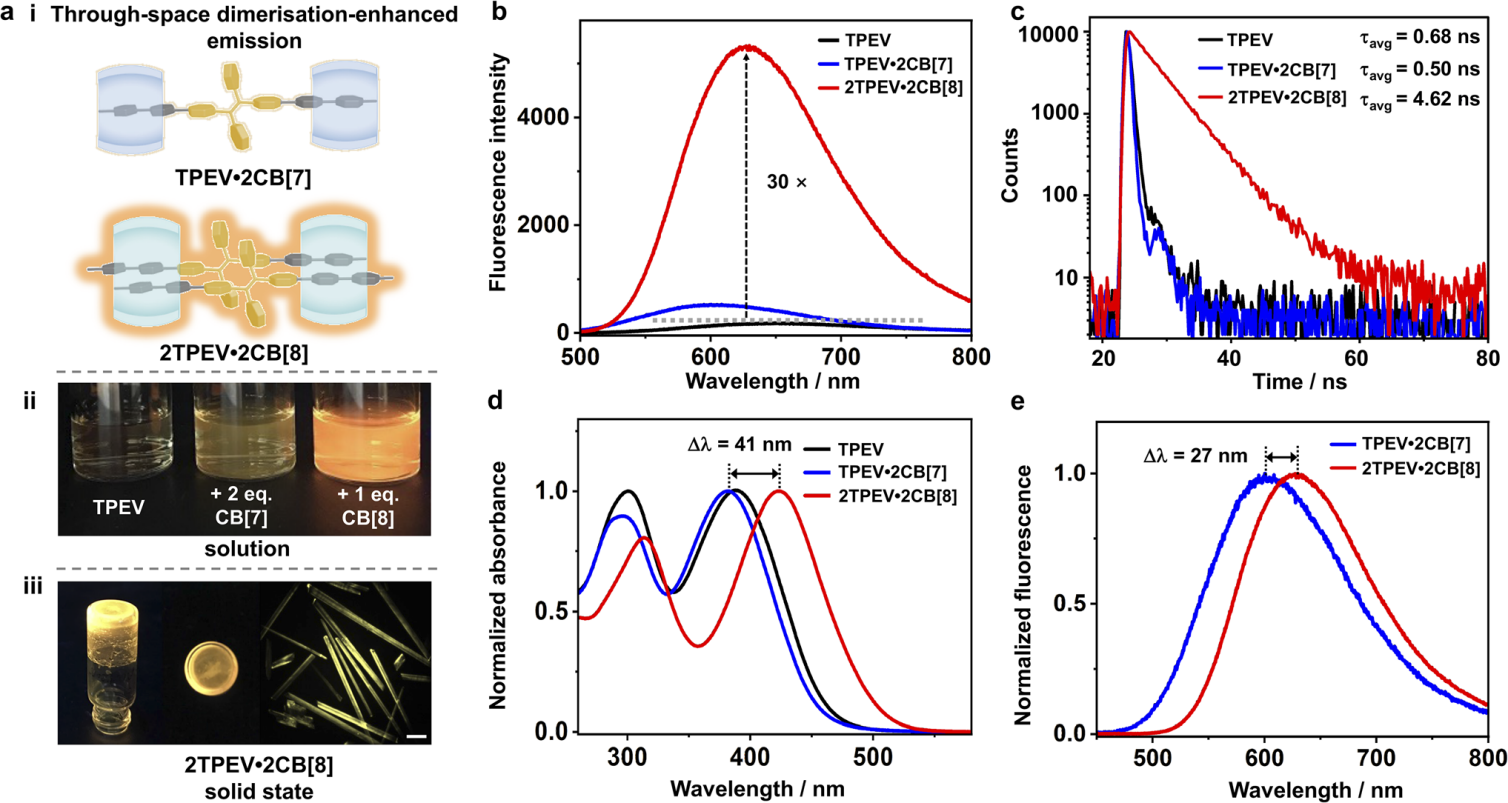
(a) (i) Schematic of the fluorescent TPEV·2CB[7] and 2TPEV·2CB[8] complexes, and photographs of (ii) aqueous solution of TPEV, TPEV·2CB[7] and 2TPEV·2CB[8] under UV irradiation, and (iii) amorphous solid and needle-like crystal (scale bar: 100 μm) of 2TPEV·2CB[8] under UV irradiation. (b) Fluorescence spectra of TPEV, TPEV·2CB[7] and 2TPEV·2CB[8] (*λ*_ex_ = 420 nm, [TPEV] = 20 μM). (c) Fluorescence decay profiles of TPEV, TPEV·2CB[7] and 2TPEV·2CB[8] along with their corresponding average lifetime (*τ*_avg_). (d) Normalised UV-Vis spectra of TPEV, TPEV·2CB[7] and 2TPEV·2CB[8] ([TPEV] = 20 μM). (e) Normalised fluorescence spectra of TPEV·2CB[7] and 2TPEV·2CB[8] (*λ*_ex_ = 420 nm, [TPEV] = 20 μM).

The above results confirm that TPEV·2CB[7] and 2TPEV·2CB[8] exist as discrete monomeric and dimeric structures, respectively, without further aggregation. The presence of CB[*n*] macrocycles within both complexes provides steric hindrance, preventing subsequent aggregation. Previously, CB[*n*]s (*n* = 7, 8) have been used to sequester fluorophores through the formation of host–guest complexes, preventing stacking and enhancing emission.^57^ However, in contrast to conventional fluorescent guests, TPE derivatives exhibit AIE as shown by the addition of isopropanol, a poor solvent, to an aqueous solution of TPEV (Fig. S21). Surprisingly, the 2TPEV·2CB[8] complex in water (without poor solvent) shows extremely intense emission behaviour even at low concentrations (10 μM) (Fig. 3a(ii)). The dimeric TPEV complex (2TPEV·2CB[8]) possesses remarkably strong emission in both dilute solution and aggregated states, spanning from amorphous to crystalline solids (Fig. 3a(iii); unfortunately, the extreme fragility of any crystals formed prevented further determination of the structure by diffraction techniques). Conformational restriction of the 2 : 2 complexation largely rigidifies the fluorophores, leading to unprecedented through-space dimerisation-enhanced emission (DEE). More importantly, the DEE phenomenon results from photophysical behaviour at the molecular level, enabling fluorescence in all states, including both solution and aggregate states. Clearly, TPEV itself is almost non-fluorescent in pure water (Fig. 3a(ii)), while the TPEV·2CB[7] complex displays a very weak fluorescence in the same condition. The weak emission of TPEV·2CB[7] in pure water is completely different from that of the CB[7] complex with planar fluorophore (Ant910Me·2CB[7]) that usually exhibits very strong emission (Fig. 1c).^56,57^ The phenyl groups in TPEV·2CB[7] are free to rotate while the phenyl groups in 2TPEV·2CB[8] are totally restricted by each other (Fig. S19), which results in a weak fluorescence of TPEV·2CB[7] and strong fluorescence of 2TPEV·2CB[8].

To gain quantitative information about the through-space DEE, steady-state absorbance and fluorescence as well as transient-state fluorescence experiments were designed and performed. As shown in Fig. 3b, steady-state fluorescence spectra confirmed the large emission enhancement of 2TPEV·2CB[8], where compared to TPEV, the fluorescence intensity of 2TPEV·2CB[8] was significantly increased by over 30 times. Notably, fluorescent titration experiments further elucidate the 1 : 1 (*n* : *n*) binding stoichiometry of the 2TPEV·2CB[8] complex (Fig. S22). Transient-state fluorescence experiments, time-correlated single photon counting (TCSPC), were performed to investigate the fluorescence decays and corresponding lifetimes of the complexes (Fig. 3c). Excited TPE dimers within the 2TPEV·2CB[8] complexes exhibited an excimer-like state that displayed an average lifetime (*τ*_avg_) of 4.62 ns, > 6*x* that of TPEV (*τ*_avg_ = 0.68 ns) alone, and ≈ 9*x* the TPEV·2CB[7] complex (*τ*_avg_ = 0.50 ns). Moreover, red shifts in both absorption (Fig. 3d, Δ*λ*_abs_ = 41 nm) and emission (Fig. 3e, Δ*λ*_em_ = 27 nm) were further observed, consistent with the shifted alignment of TPEV in the dimer due to the bulky volume of TPE. We further measured the solid-state fluorescence spectra of all three systems (Fig. S23), which showed that 2TPEV·2CB[8] remains the most emissive in the solid state, consistent with its behaviour in solution. This highlights that its emission originates from supramolecular dimerisation at the molecular level, rather than being state-dependent.

To evaluate the photophysical properties of 2TPEV·2CB[8], a series of related parameters were obtained and summarised as shown in Table 1. Typically, unbound TPEV exhibits a low fluorescence quantum yield (*ϕ*_F_ = 4%). Through host–guest complexation with CB[7], the fluorescence quantum yield of TPEV was increased to 6%. More impressively, after complexation with CB[8], the fluorescence quantum yield of TPEV was significantly increased to 83%, > ×20 higher than TPEV itself. The rate constants of the radiative (*k*_r_) and non-radiative (*k*_nr_) pathways were further calculated using the fluorescence lifetime (*τ*_avg_) and quantum yield (*ϕ*_F_) values. The radiative rate constant of 2TPEV·2CB[8] (*k*_r_ = 180.3 μs^−1^) is of the same order of magnitude as that of TPEV·2CB[7] (*k*_r_ = 128.0 μs^−1^), with only a minor difference between the two. However, when compared with TPEV·2CB[7] (*k*_nr_ = 1872.0 μs^−1^), the non-radiative rate constant of 2TPEV·2CB[8] (*k*_nr_ = 36.2 μs^−1^) is nearly two orders of magnitude lower. This reduction is attributed to the significantly longer lifetime (*τ*_avg_ = 4.62 ns) and the high fluorescence quantum yield (*ϕ*_F_ = 83%) of 2TPEV·2CB[8]. Meanwhile, the molar absorption coefficient (*ε*) of 2TPEV·2CB[8] is slightly higher than TPEV·2CB[7] (Table 1), leading to enhanced emission brightness (*ε* × *ϕ*_F_ = 29.2) in aqueous media. The high fluorescence efficiency of 2TPEV·2CB[8] is on account of the substantial suppression of intra-complex motions within the bulky TPE dimer, which greatly minimise non-radiative relaxation pathways.

**Table 1:** Summary of photophysical parameters of Ant910Me and TPEV as well as their host–guest complexes at 298 K

Species	*λ*_abs_ (nm)	*λ*_em_ (nm)	Δ*ν* (cm^−1^)	*τ*_1_ (ns)	*τ*_2_ (ns)	*τ*_avg_ (ns)	*ϕ*_F_ a (%)	*k*_nr_ (μs^−1^)	*k*_r_ (μs^−1^)	*ε* b (10^4^ M^−1^ cm^−1^)	Brightness^c^ (10^3^ M^−1^ cm^−1^)
Ant910Me	419	595	7060	1.66 (96%)	9.50 (4%)	1.97	4	486.4	20.3	0.9	0.4
Ant910Me·2CB[7]	409	537	5828	—	—	8.60	85	17.4	98.8	1.2	10.3
2Ant910Me·2CB[8]	469	578	4021	—	—	12.6	81	15.1	64.3	1.3	10.8
TPEV	389	651	10 346	0.55 (96%)	3.77 (4%)	0.68	4	1408.8	61.8	2.9	1.2
TPEV·2CB[7]	381	600	9579	0.37 (96%)	3.71 (4%)	0.50	6	1872.0	128.0	3.1	2.0
2TPEV·2CB[8]	422	627	7748	4.42 (99%)	22.72 (1%)	4.62	83	36.2	180.3	3.5	29.2

a *ϕ*_F_: Fluorescence quantum yield.

b *ε*: Molar absorption coefficient at *λ*_abs_.

c Brightness = *ε* × *ϕ*_F_

A comprehensive comparison of the photophysical properties between TPEV (bulky, 3D shaped core) and Ant910Me (planar core) provides additional insights (Table 1). As a discrete monomer, TPEV·2CB[7] exhibits only weak fluorescence emission, whereas Ant910Me·2CB[7] shows strong fluorescence emission. This contrast clearly demonstrates that (1) CB[7] can prevent the aggregation of Ant910Me, thereby significantly inhibiting the ACQ effect; and (2) the TPEV monomer displays weak emission, where non-radiative pathways dominate. The discrete 2TPEV·2CB[8] dimer, shows strong fluorescence, which differs from the strong emission behaviour of 2Ant910Me·2CB[8]. The latter exhibits strongly coupled and effectively delocalised π electrons in its ground state, on account of the planar nature of the linker. This pre-organised π-stacked ground-state dimer is excited into an excimer state as a pre-coupled entity without the need for additional diffusion controlled processes after photoexcitation, and shows a significant emission colour difference with Ant910Me·2CB[7] (Δ*λ*_em_ = 41 nm). In contrast, the large TPE groups in 2TPEV·2CB[8] experience significant steric crowding, squeezed by CB[8] macrocycles at both ends. This greatly restricts molecular motion and suppresses the non-radiative decay pathways, resulting in AIE from the 2TPEV·2CB[8] dimer.

## Conclusions

In conclusion, the initial stage of aggregation for AIE fluorophores was systematically studied using a TPE derivative (TPEV) as a model. Our results confirm that two AIE fluorophores are the minimum required for through-space aggregation-induced luminescence. Host–guest interactions with CB[7] and CB[8] macrocycles enabled the formation of a discrete TPEV monomer and a tightly aggregated dimer, respectively. While the TPEV monomer is weakly emissive, the dimer exhibits enhanced photophysical properties, including elongated fluorescence lifetime, increased emission intensity, high quantum yield, with limited redshifts in both absorption and emission. The 2TPEV·2CB[8] dimer fluoresces across all states: dilute and concentrated solutions as well as amorphous and crystalline solids. This work provides fundamental insights into the initial aggregation of AIE fluorophores, which addresses a critical question (how many fluorophores are required to achieve AIE?) that has undoubtedly persisted in the field, receiving little attention until now due to the lack of a suitable molecular model.^34^ Additionally, our findings offer a strategy for designing highly emissive materials for applications in chemosensors, bioimaging, and optoelectronics.

## Author contributions

Conceptualisation: T. X., Z. H., O. A. S. formal analysis: T. X., Z. H., G. W., J. A. M, O. A. S funding acquisition: T. X., G. W., O. A. S. investigation: T. X., Z. H., L. Z., S. J., H. Q. project administration: J. A. M, O. A. S supervision: T. X., G. W., O. A. S writing – original draft: T. X., Z. H., X. C., G. W. writing – review & editing: T. X., Z. H., X. C., G. W., J. A. M., O. A. S.

## Conflicts of interest

There are no conflicts to declare.

## Supplementary Material

SC-OLF-D5SC04053A-s001

## Data Availability

The data supporting this article have been included as part of the SI. Supplementary information is available. See DOI: https://doi.org/10.1039/d5sc04053a.
