# Supplementary material for: How many fluorophores are required to achieve AIE?
Source: Chem Sci. 2025 Oct 21;16(46):22058–64. doi: 10.1039/d5sc04053a (PMC12539275; doi:10.1039/d5sc04053a)
Supplement: SC-016-D5SC04053A-s001 [file SC-016-D5SC04053A-s001.pdf]

Supplementary Information (SI) for Chemical Science.
This journal is © The Royal Society of Chemistry 2025

Supporting Information

How many fluorophores are required to achieve AIE?

Tangxin Xiao,[‡a,b] Zehuan Huang,[‡a], Liangliang Zhang,[b] Shuang Jin,[c] Xiaoyi Chen,[a] Hongwei Qian,[b] Guanglu Wu,[c] Jade A. McCune[a] and Oren A. Scherman[*a]

[a] *Melville Laboratory for Polymer Synthesis, Yusuf Hamied Department of Chemistry, University of Cambridge, Cambridge CB2 1EW, United Kingdom. E-mail: oas23@cam.ac.uk*

[b] *Jiangsu Key Laboratory of Advanced Catalytic Materials and Technology, School of Petrochemical Engineering, Changzhou University, Changzhou 213164, P. R. China*

[c] *State Key Laboratory of Supramolecular Structure and Materials, College of Chemistry, Jilin University, Changchun 130012, P. R. China*

[‡]These authors contributed equally to this work

Table of Contents

SI-1. Materials & instrumentation 2

SI-2. Synthesis and characterisation of TPEV 4

SI-3. Through-space dimerisation of TPEV via CB[8] 9

SI-4. Supramolecular complexation of TPEV and CB[7] 13

SI-5. Conformational comparison of the monomer and dimer. 16

SI-6. Additional photophysical data 17

References 19

SI-1. Materials & instrumentation

Materials. Unless otherwise stated, all the chemicals in this research were purchased from Sigma Aldrich and directly used without further purification: 1,2-bis(4-bromophenyl)-1,2-diphenylethene (Adamas reagent, 98%), pyridine-4-boronic acid (Adamas reagent, 98%), tetrakis(triphenylphosphine)palladium (Adamas reagent, 99%), 1-chloro-2,4-dinitrobenzene (98%, Alfa Aesar), *p*-toluidine (99%), acetonitrile (HPLC, 99.9%), ethanol (absolute, 99.8, HPLC), *N*,*N*-dimethylformamide (DMF, 99%), dichloromethane (99%), acetone (ACS reagent, 99.5%), diethyl ether (ACS reagent, 99%), deuterium oxide (D_2O, D 99.8 atom %), dimethyl sufoxide-d_6 (DMSO-d_6, D 99.9 atom %). Cucurbit[7]uril (CB[7]) and cucurbit[8]uril (CB[8]) were synthesised and isolated in 100 gram scale from the mixture of cucurbit[n]uril (CB[n]) derivatives using a previously reported protocol.[S1] Milli-Q water was simply obtained from a Milli-Q Integral Water Purification System (18.2 MW·cm). Unless otherwise noted, all the sample solutions for characterisation were prepared in D_2O or Milli-Q H_2O under heating and ultrasonication.

Nuclear Magnetic Resonance Spectroscopy (NMR). ^{1}H NMR, ^{13}C NMR, COSY, DOSY, and NOESY spectra were acquired in deuterated water (D_2O) and recorded on either a Bruker AVANCE 400 (400 MHz) or a Bruker AVANCE 500 with TCI Cryoprobe system (500 MHz) being controlled by TopSpin2. DOSY experiments were carried out using a modified version of the Bruker sequence ledbpgp2s involving, typically, 32 scans over 16 steps of gradient variation from 10% to 80% of the maximum gradient. NOESY experiments were carried out using a standard pulse sequence 'noesygpphpp' with a 2 s relaxation delay and a 1000 ms mixing time. The peaks were calibrated against the residual proton signal or natural abundance carbon resonance of the used deuterated solvent from tetramethylsilane (TMS) as the internal standard. The chemical shifts δ are indicated in ppm and the coupling constants J in Hz.

The multiplicities are given as s (singlet), d (doublet), dd (doublet of doublets), t (triplet), and m (multiplet).

Electrospray Ionisation Mass Spectrometry (ESI-MS). ESI-MS spectra were acquired on a Thermo Fisher Q Exactive Orbitrap mass spectrometer with a nano-spraying ionisation source, using borosilicate emitters as the one-off needles to inject the sample solutions into the MS apparatus. ESI-MS experiments were performed in the positive ion mode at the *m/z* range from 150 - 2000 under the working temperature at 320 °C and the capillary voltage of 1.5 kV. The *m/z* value was calibrated in advance by a standard test sample before all the MS characterisation. All the sample solutions used in MS experiments were prepared in Milli-Q water at 0.5 mM, and all the obtained data were analysed in Origin 10.0 software.

Isothermal titration calorimetry (ITC). ITC experiments were conducted on a Malvern MicroCal Auto-ITC200 apparatus at 298.15 K in Milli-Q H_2O. In a typical ITC, the host molecule (CB[7] or CB[8]) was placed in the sample cell, and the guest molecule was put in the injection syringe with a concentration of about ten times the concentration of the host. One titration experiment consisted of one injection of 0.6 μL and 32 consecutive injections of 1.2 μL with 90 s intervals between injections. The first one or two data points were removed before data analysis as they may contain contamination, and the resultant ITC curves were fitted by one set of sites model within Malvern MicroCal Analysis Centre software to gain thermodynamic information.

Spectroscopic Measurements. UV-Vis absorbance and steady-state fluorescence spectra were acquired and recorded at room temperature on a Duetta-Fluorescence and Absorbance Spectrometer (HORIBA Scientific).

Lifetime and quantum yield of luminescence. The luminescence lifetime and quantum yield were measured using an Edinburgh Instruments FLS920 spectrofluorometer. A 372.2 nm laser (EPL-375 model) was employed as the excitation

source. The luminescence lifetime was determined using the time-correlated single-photon counting (TCSPC) method and corresponding accessories, while the quantum yield was recorded using an integrating sphere module. All measurements were conducted using a 1 mm × 10 mm quartz cuvette with an excitation path length of 10 mm. The temperature was maintained at 298 K, and the sample volume was 500 μL. The concentration of the guest molecule in the sample was 20 μM, and the solvent used was deuterium oxide (D_2O).

SI-2. Synthesis and characterisation of TPEV

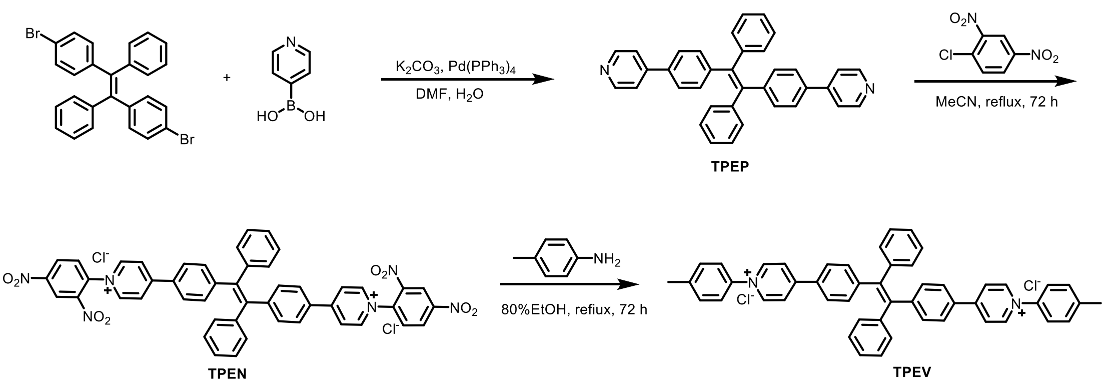

Scheme S1 Overview of synthetic route to prepare TPEV.

Synthesis of TPEP. Compound TPEP was synthesised according to a literature report by Suzuki cross-coupling of bis(4-bromophenyl)-1,2-diphenylethene and pyridine-4-boronic acid.[S2] Under a nitrogen atmosphere, 1,2-bis(4-bromophenyl)-1,2-diphenylethene (1.76 g, 3.5 mmol), 4-pyridylboronic acid (3.50 g, 28.0 mmol), potassium carbonate (14.60 g, 105.0 mmol), tetrakis(triphenylphosphine)palladium(0) (0.29 g, 0.25 mmol), *N*,*N*-dimethylformamide (DMF, 90 mL), and deionized water (30 mL) were sequentially added to a round-bottom flask. The mixture was heated to reflux and stirred under a continuous nitrogen flow for 48 h. Reaction progress was monitored by thin-layer chromatography (TLC). Upon completion, the reaction mixture was poured into water and extracted with dichloromethane (3 × 30 mL). The combined organic extracts were washed with saturated aqueous NaCl solution (3 × 50 mL), dried

over anhydrous sodium sulfate, and concentrated under reduced pressure. The crude product was purified by silica gel column chromatography using dichloromethane/methanol (200:1, v/v) as the eluent to afford the target trans-compound TPEP (0.81 g, 1.6 mmol) as a white solid in 46% yield. ^{1}H NMR (300 MHz, DMSO-d_6, 298 K): δ (ppm) = 8.59 (d, J = 6 Hz, 4H, Py-*H*), 7.68-7.63 (m, 8H, Ar-*H*), 7.21-7.06 (m, 14H, Ar-*H*).

Synthesis of TPEV. TPEV was prepared by the transformation of the pryidin-4-yl groups of TPEP into arylpyridinium salts through a Zincke reaction: 1-Chloro-2,4-dinitrobenzene (78.0 mg, 0.39 mmol, 3.5 equiv.) and TPEP (51.4 mg, 0.11 mmol) were refluxed in acetonitrile (5 mL) at 82 °C for 72 h under nitrogen atmosphere. The mixture was filtered and the resultant cake was washed with dichloromethane (5 mL). The red product was dried and directly used for next step. Such intermediate product (72 mg, 0.088 mmol, 1.0 equiv.) and *p*-toluidine (20.6 mg, 0.2 mmol, 2.2 equiv.) were refluxed in ethanol (8 mL) at 78 °C for 12 h under nitrogen atmosphere. The mixture was filtered and the filtrate was evaporated to afford the crude product, which was re-dissolved into acetone (100 mL) and washed by sonication and filtration. The obtained raw material was further washed with ethanol and dried in vacuum oven to give pure TPEV as a yellow solid in 85 % yield (49.7 mg, 0.072 mmol). ^{1}H NMR (400 MHz, D_2O, 298.15 K): δ (ppm) = 8.90 (d, J = 5.8 Hz, 4H), 8.30 (d, J = 7.6 Hz, 4H), 7.75 (d, J = 8.1 Hz, 4H), 7.53 (d, J = 8.2 Hz, 4H), 7.48 (d, J = 6.3 Hz, 4H), 7.34 (d, J = 9.0 Hz, 4H), 7.23-7.13 (m, 10H), 2.4 (s, 6H). ^{13}C NMR (100 MHz, D_2O, 298.15 K): δ (ppm) = 147.76, 143.71, 142.65, 142.31, 141.54, 139.89, 132.47, 132.36, 131.80, 131.27, 130.83, 128.21, 127.61, 127.36, 124.40, 123.50, 20.24. HR-ESI-MS for [TPEV-2Cl]$^{2+}$: calc. m/z = 334.1590, found m/z = 334.1585.

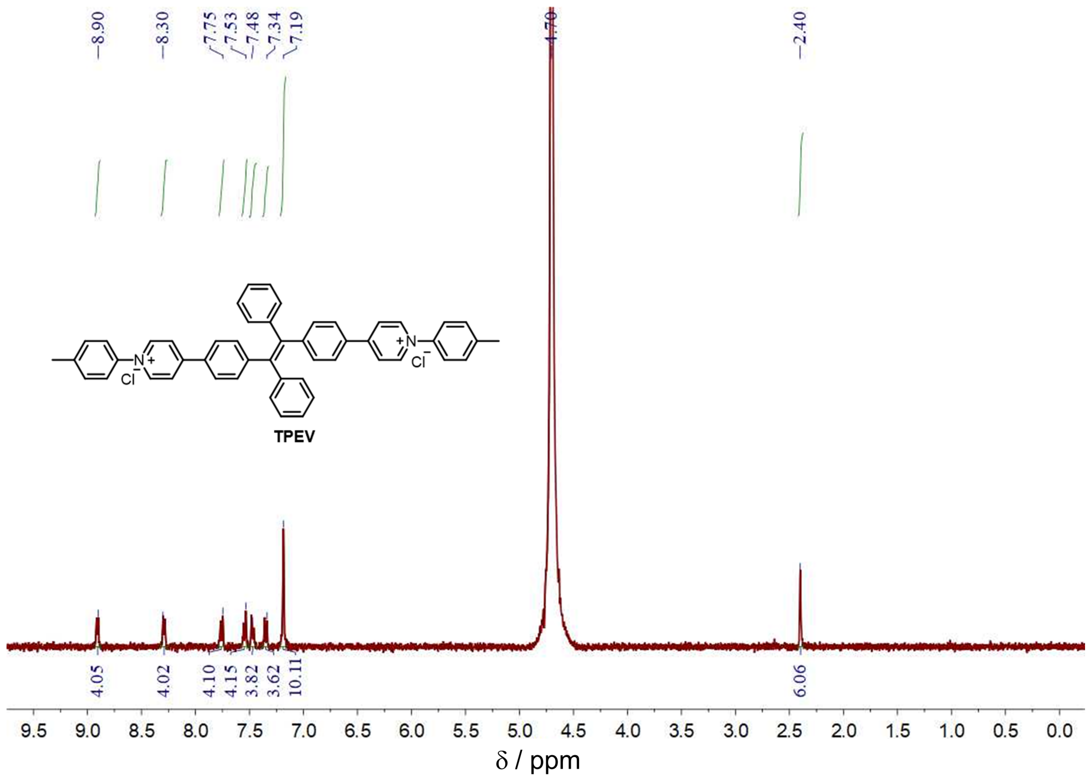

Figure S1. ^{1}H NMR (400 MHz, D_2O, 298.15 K) spectrum of TPEV.

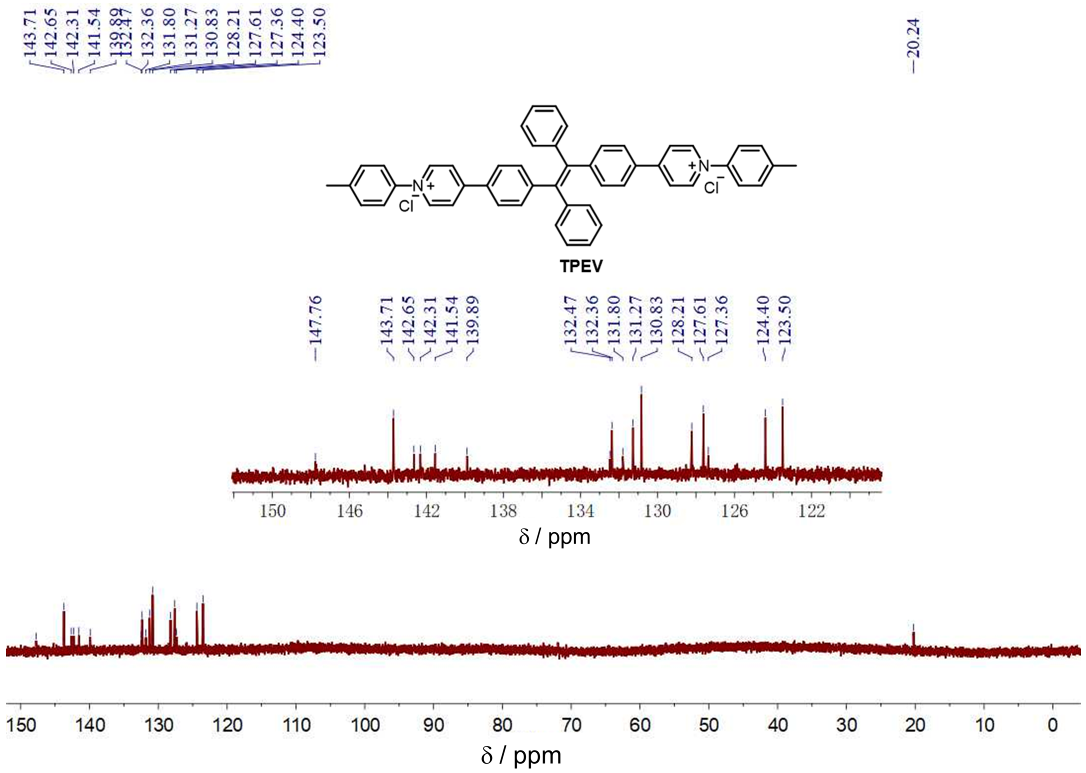

Figure S2. ^{13}C NMR (126 MHz, D_2O, 298.15 K) spectrum of TPEV.

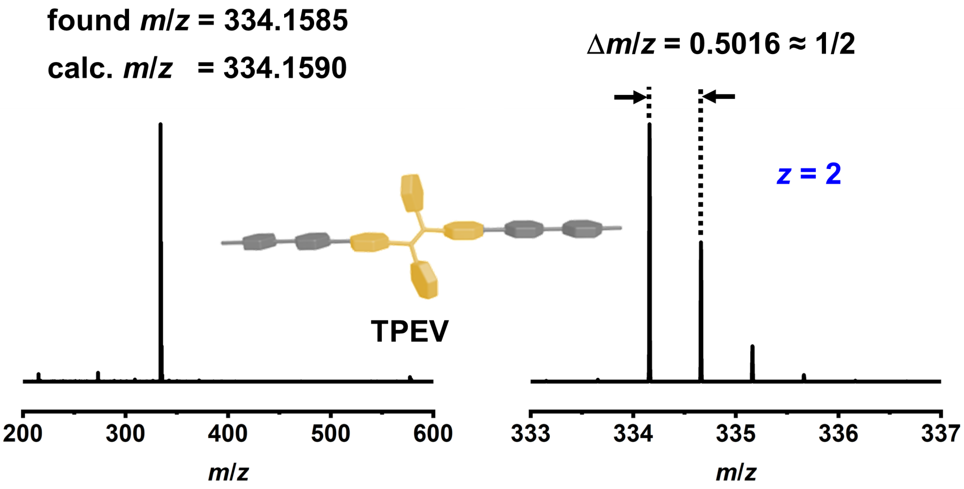

Figure S3. HR-ESI-MS spectra of TPEV.

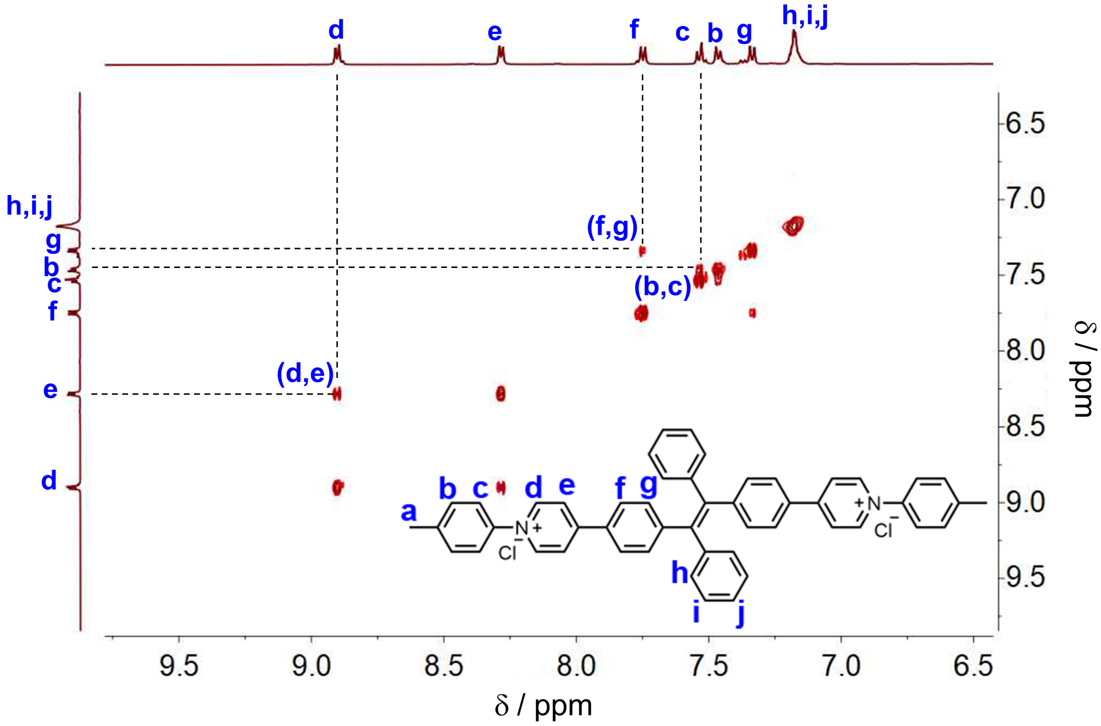

Figure S4. ^{1}H-^{1}H COSY (500 MHz, D_2O, 298.15 K) spectrum of TPEV.

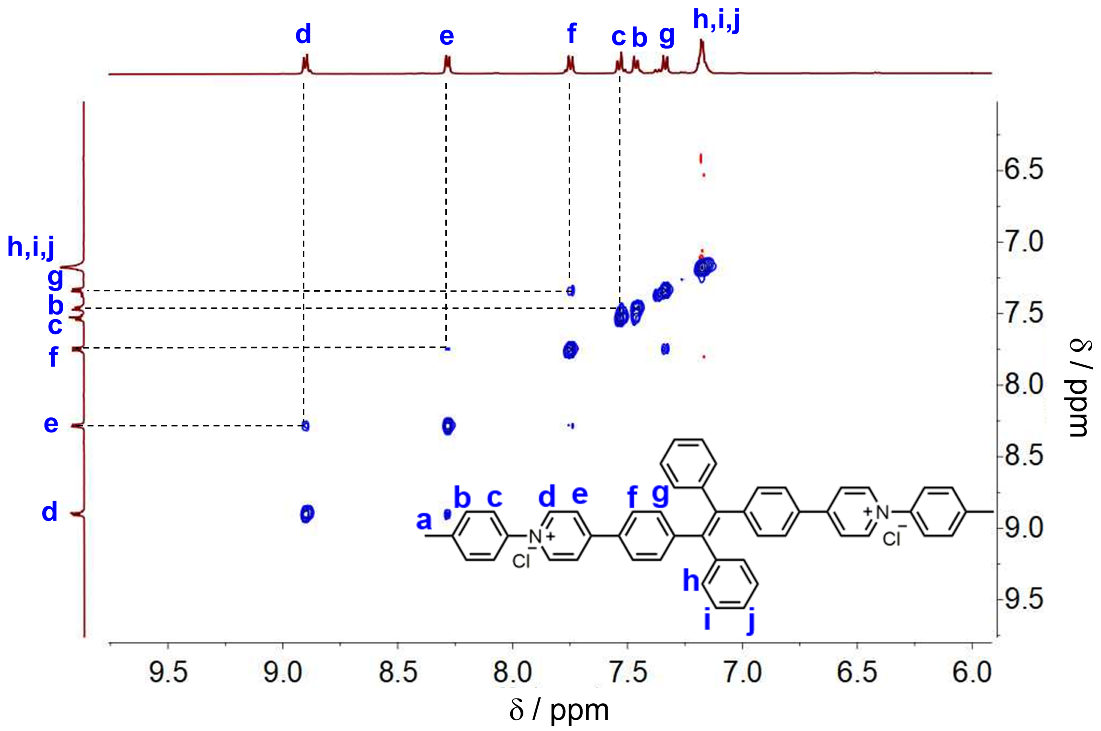

Figure S5. ${}^{1}H$-${}^{1}H$ NOESY (500 MHz, D_2O, 298.15 K) spectrum of TPEV.

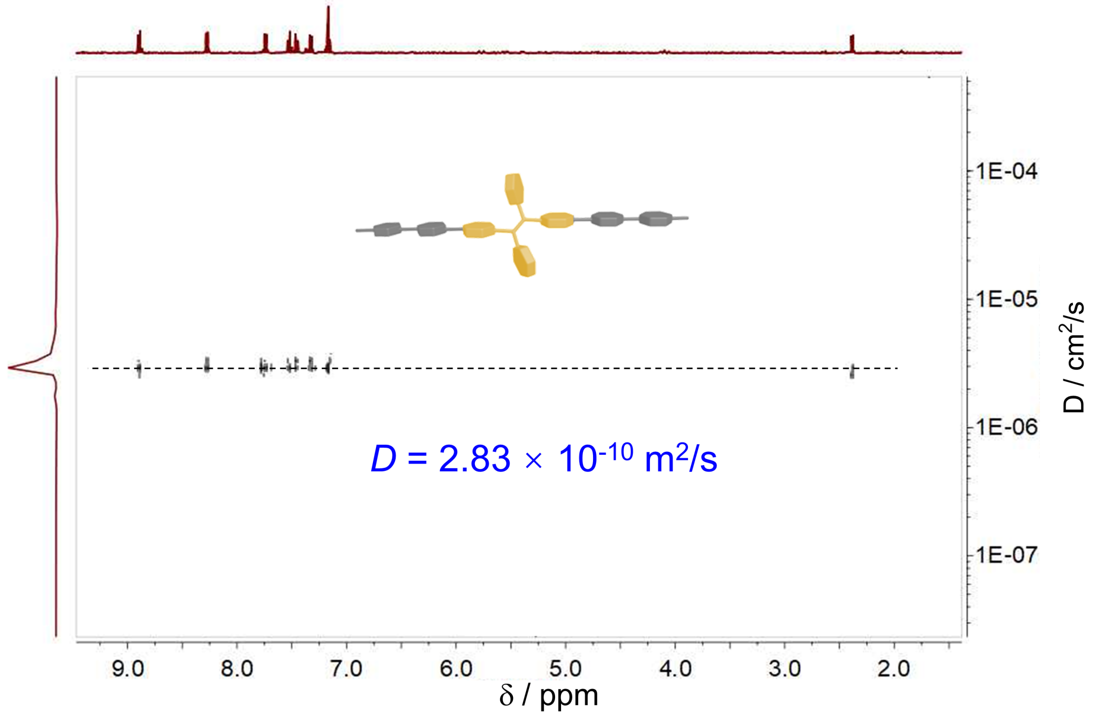

Figure S6. DOSY (400 MHz, D_2O, 298.15 K) spectrum of TPEV.

SI-3. Through-space dimerisation of TPEV via CB[8]

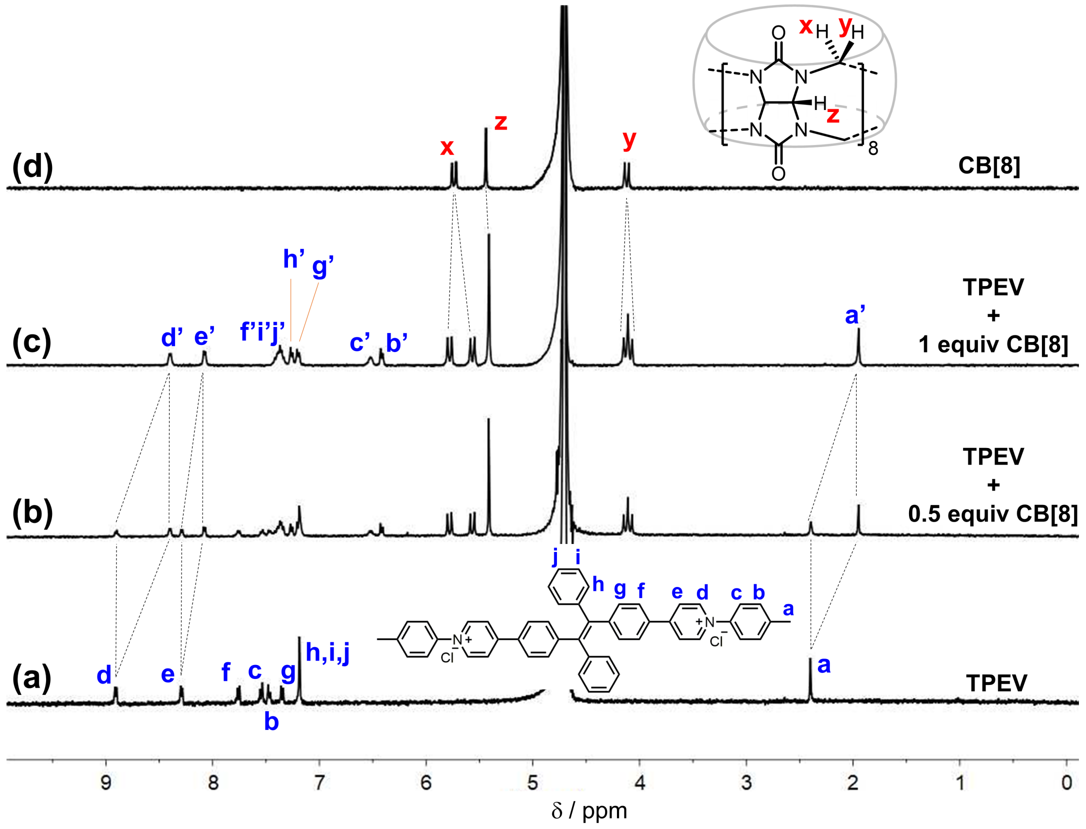

Figure S7. ^{1}H NMR (400 MHz, D_2O, 298.15 K) spectra of (a) TPEV, (b) TPEV with 0.5 eq. of CB[8], (c) TPEV with 1 equiv. of CB[8], and CB[8]. [TPEV] = 0.2 mM.

When 1 equiv. CB[8] was added to an aqueous solution of TPEV, the protons from the tolyl and pyridinium moieties exhibited significant upfield shifts (tolyl -- Δδ, H_a: +0.44 ppm, H_b: +1.05 ppm and H_c: +1.02 ppm; pyridinium -- Δδ, H_d: +0.51 ppm and H_e: +0.21 ppm), respectively. Meanwhile, the protons (H_x and H_y) on the portal of CB[8] split into two sets of peaks, suggesting the formation of a host-guest complex with tolyl and pyridinium moieties located inside the CB[8] cavity. Moreover, only one set of peaks for TPEV protons were observed in the equimolar mixture of TPEV and CB[8], indicating that the chemical environment within the 2:2 complex is symmetric. As TPEV is a ditopic pyridinium derivative with a large TPE moiety in its core, its bulky volume could impede the slippage of CB[8] along the guest axis.

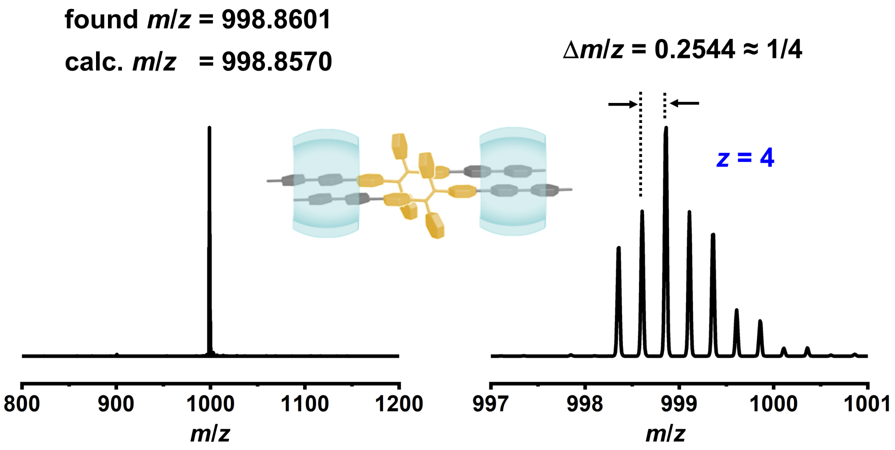

Figure S8. HR-ESI-MS spectra of 2TPEV·2CB[8].

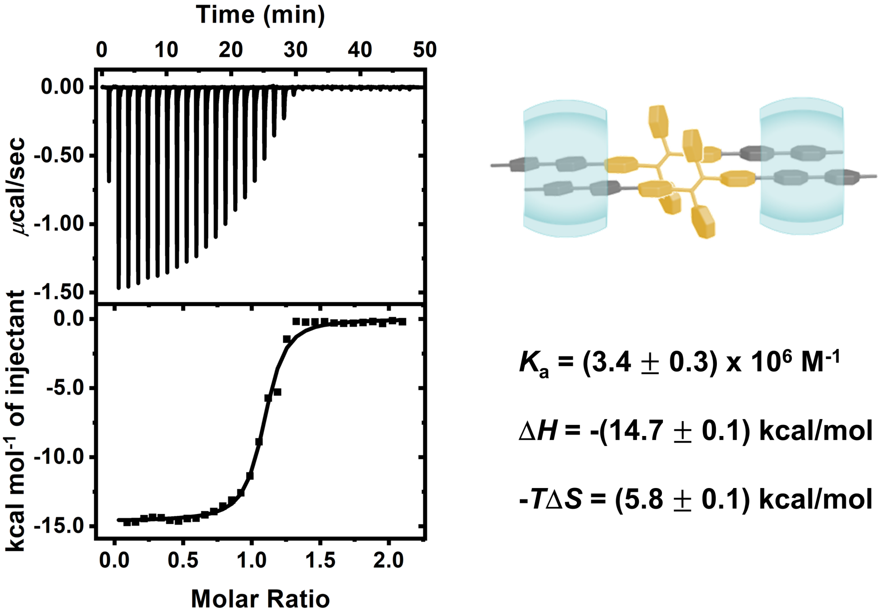

Figure S9. ITC titration plot and fitted curve obtained through titration of TPEV (0.5 mM) into CB[8] (0.05 mM) at 298.15K in pure water.

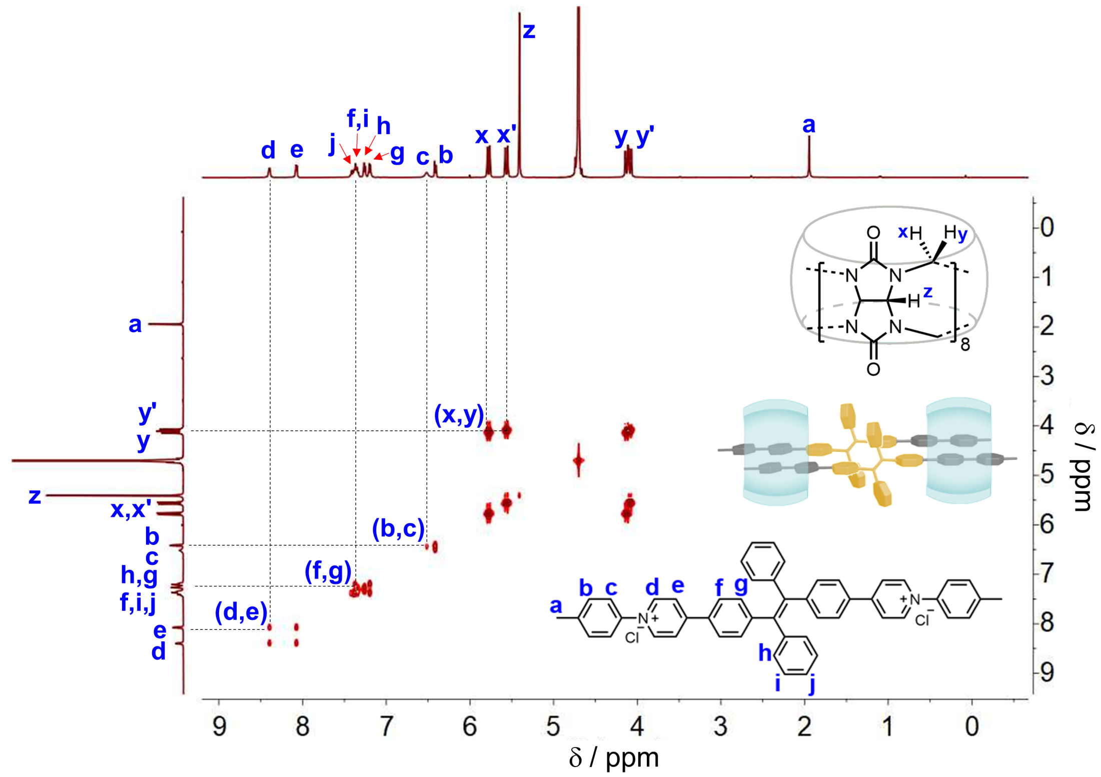

Figure S10. ^{1}H-^{1}H COSY (500 MHz, D_2O, 298.15 K) spectrum of 2TPEV·2CB[8].

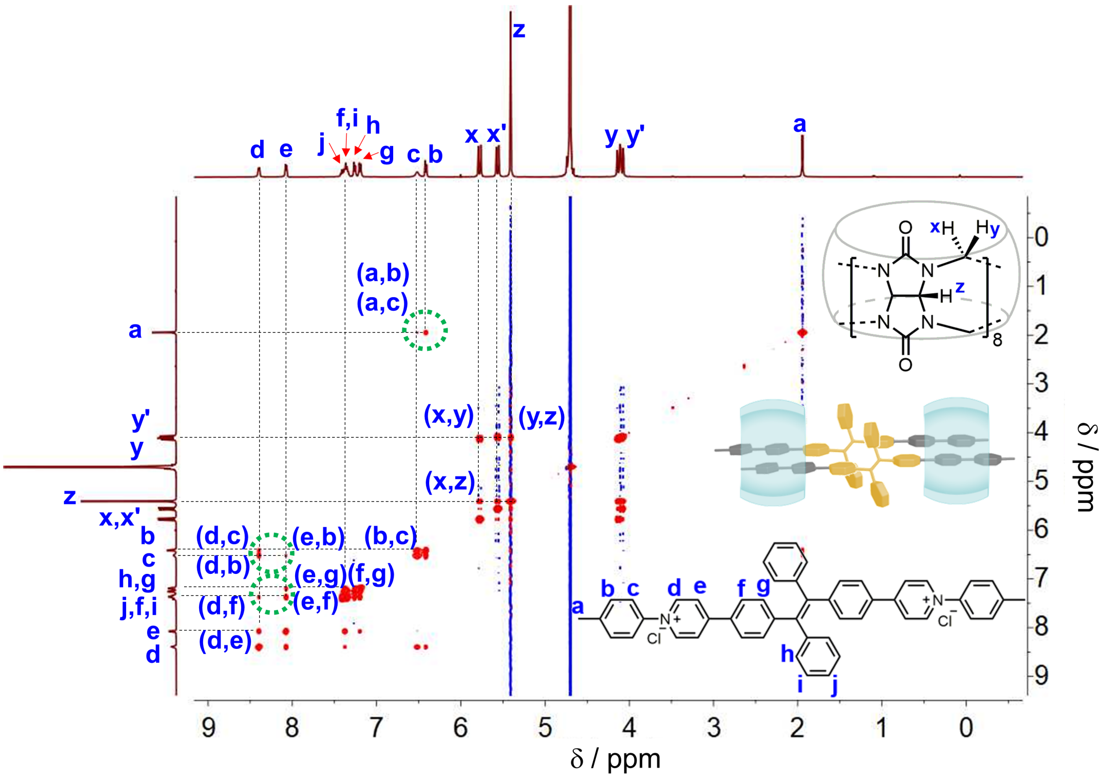

Figure S11. ^{1}H-^{1}H NOESY (500 MHz, D_2O, 298.15 K) spectrum of 2TPEV·2CB[8].

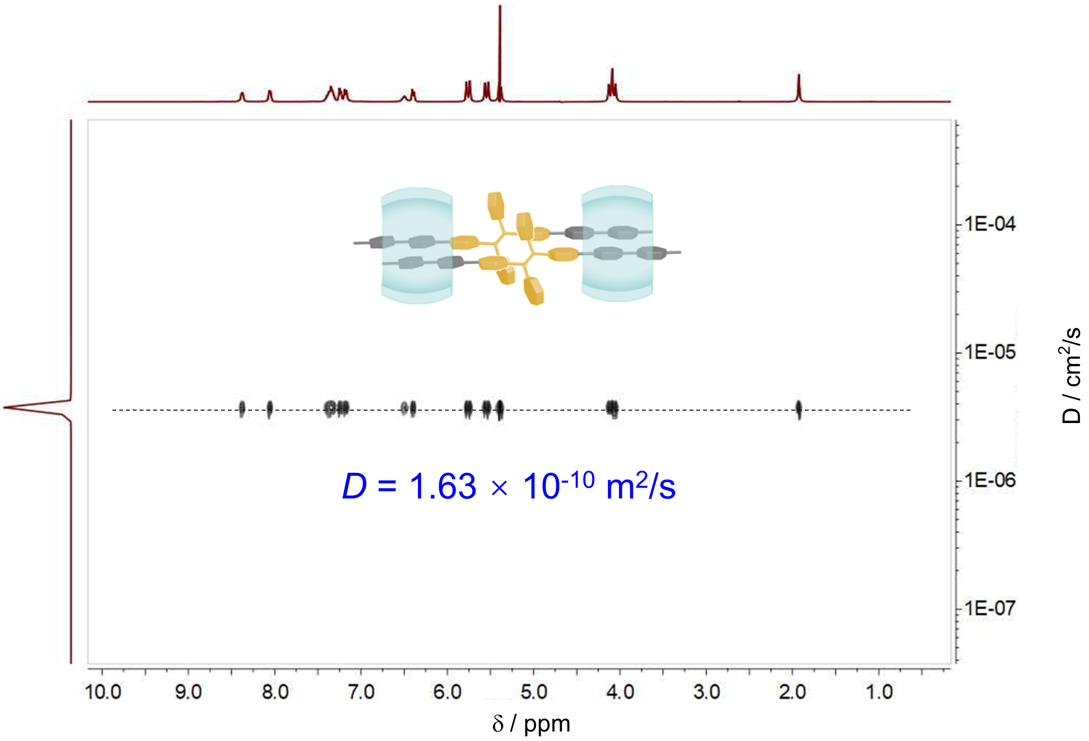

Figure S12. DOSY (400 MHz, D_2O, 298.15 K) spectrum of 2TPEV·2CB[8].

SI-4. Supramolecular complexation of TPEV and CB[7]

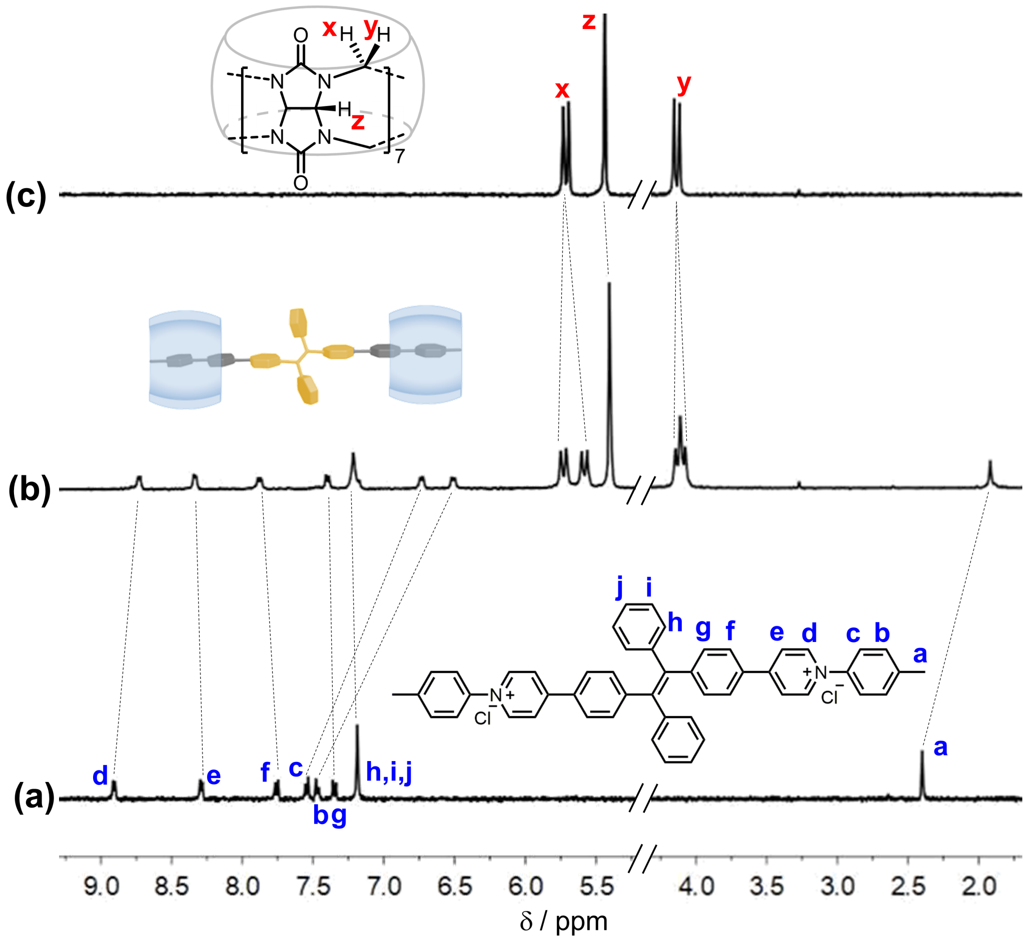

Figure S13. ^{1}H NMR (400 MHz, D_2O) spectra of (a) TPEV, (b) TPEV with 2 equiv. of CB[7], and CB[7]. [TPEV] = 0.2 mM.

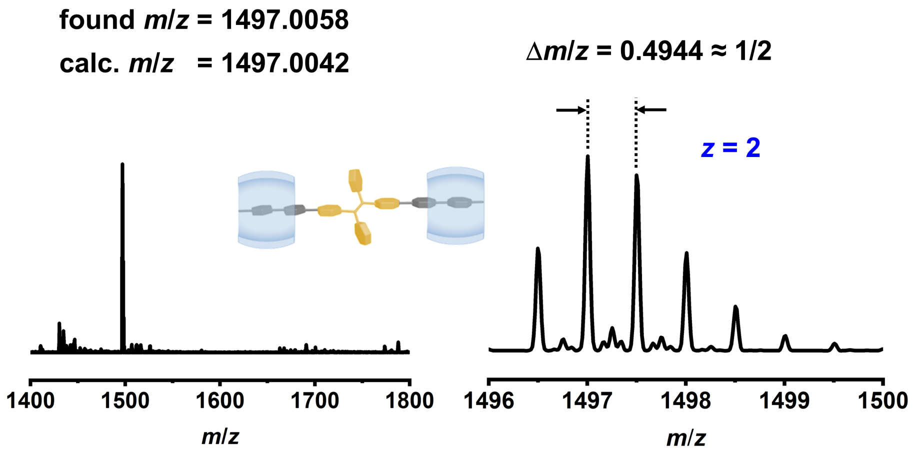

Figure S14. HR-ESI-MS spectra of TPEV·2CB[7].

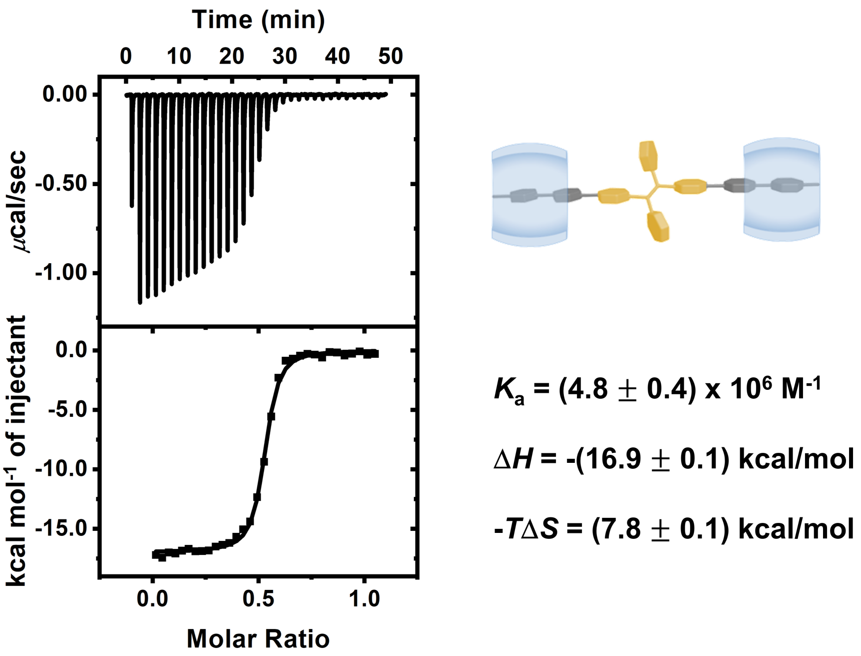

Figure S15. ITC titration plot and fitted curve obtained through titration of TPEV (0.5 mM) into CB[7] (0.1 mM) at 298.15 K in pure water.

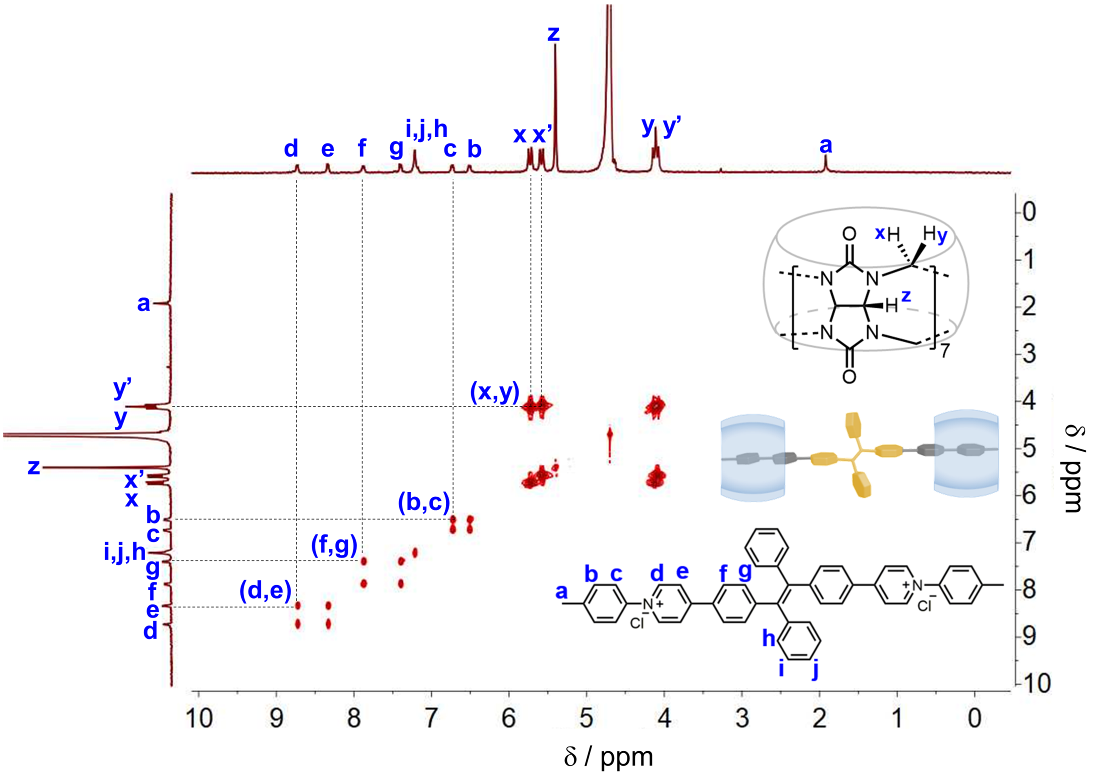

Figure S16. ^{1}H-^{1}H COSY (500 MHz, D_2O, 298.15 K) spectrum of TPEV·2CB[7].

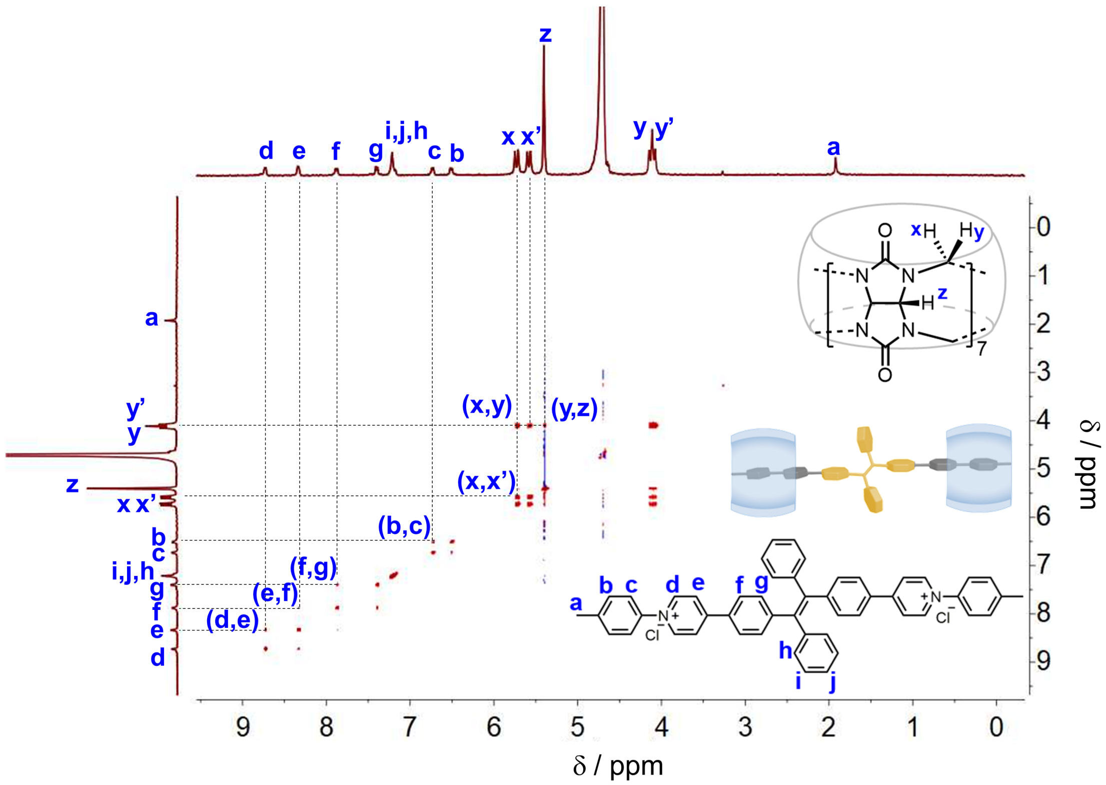

Figure S17. ^{1}H-^{1}H NOESY (500 MHz, D_2O, 298.15 K) spectrum of TPEV·2CB[7].

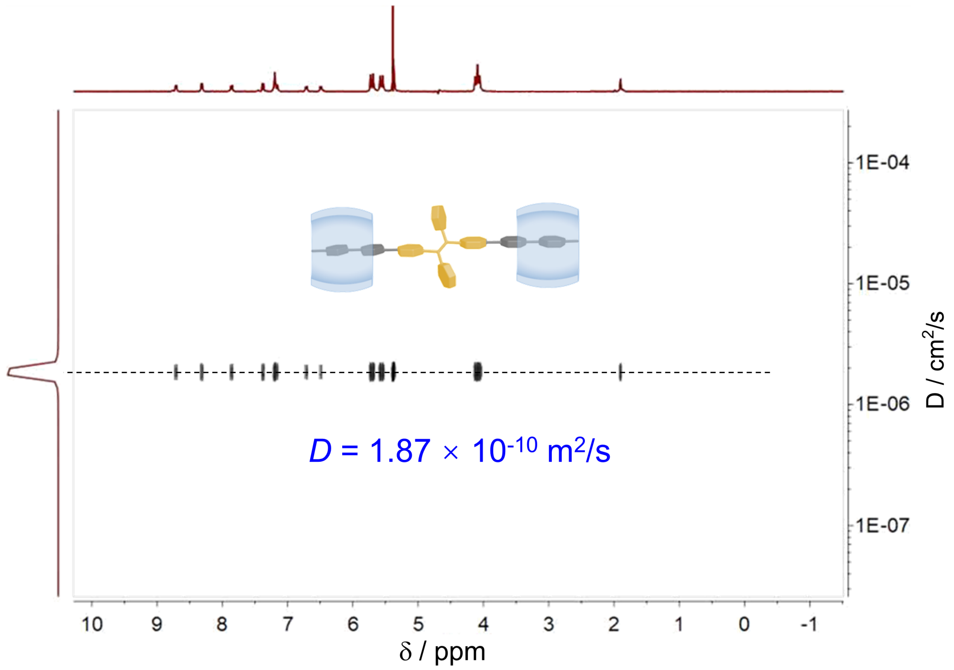

Figure S18. DOSY (400 MHz, D_2O, 298.15 K) spectrum of TPEV·2CB[7].

SI-5. Conformational comparison of the monomer and dimer.

Table S1 Summary of chemical shift change (Δδ) of protons in TPEV upon the addition of CB[7] and CB[8].

Species	H_a	H_b	H_c	H_d	H_e	H_f	H_g	H_h	$H_{i,j}$
TPEV·2CB[7]	+0.47	+0.97	+0.81	+0.17	-0.04	-0.12	-0.04	0	-0.01
2TPEV·2CB[8]	+0.44	+1.05	+1.02	+0.51	+0.21	+0.39	+0.16	-0.08	-0.19

Note: "+" represents upfield shift, and "-" represents downfield shift

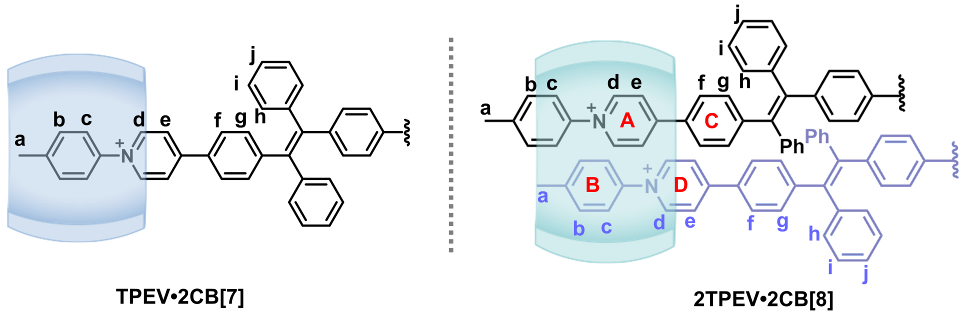

Figure S19. Illustration of the location of CB[7] or CB[8] macrocycles on the axis of TPEV. Notably, charge-transfer interaction should be occurred in 2TPEV·2CB[8] between aryl rings: A-B, and C-D.

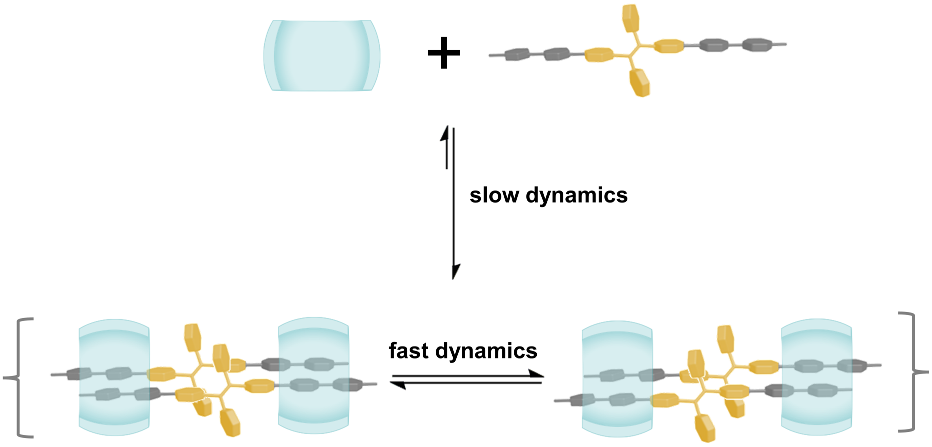

Figure S20. Illustration of the slow dynamics in the host-guest complexation process and the fast dynamics in the intra-complex conversion.

According to the ^{1}H NMR results, only one set of proton peaks was observed for TPEV in 2TPEV·2CB[8], showing that a fast dynamic conversion exists (Figure S20), which is consistent with our previous report.[S3] More in-depth information about the

conformations of the monomeric and dimeric structures was gained through comparison of their changes in chemical shifts before and after complexation (Table S1). Some interesting phenomena were observed: (1) the Δδ values of $H_{b,c}$ from tolyl moiety and $H_{d,e}$ from pyridinium moiety in 2TPEV·2CB[8] are dramatically larger than that in TPEV·2CB[7]; (2) H_f and H_g from the phenylene groups of 2TPEV·2CB[8] showed upfield shift (Δδ, H_f: +0.39 ppm and H_g: +0.16 ppm), opposite to TPEV·2CB[7]; (3) $H_{h\text{-}j}$ from the phenyl group of 2TPEV·2CB[8] showed substantial downfield shifts (Δδ, H_h: -0.08 ppm and $H_{i,j}$: -0.19 ppm). Charge-transfer interactions may occur between electron donor-acceptor pairs (A-B and C-D in Figure S19) of the pyridinium group (A) and the tolyl group (B), as well as the phenylene group (C) and pyridinium group (D) from the adjacent counterpart. As charge-transfer interaction usually induces strong shielding effect, the upfield shifts of H_f and H_g should be on account of the charge-transfer interaction rather than the shielding effect from the CB[8] cavity.

SI-6. Additional photophysical data

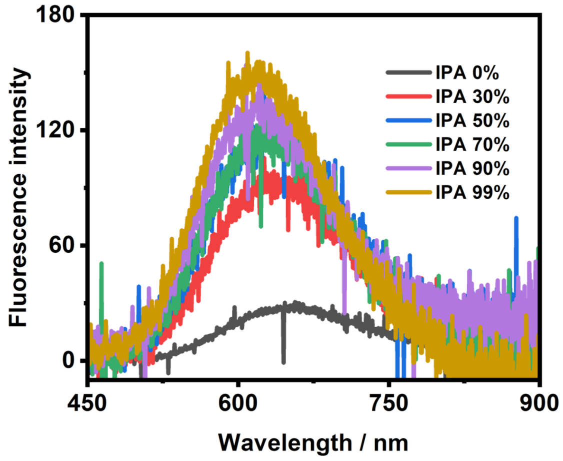

Figure S21. Fluorescence spectra of TPEV in mixed H_2O and isopropanol (IPA) solvent. [TPEV] = 2×10^{-5} M^{-1}

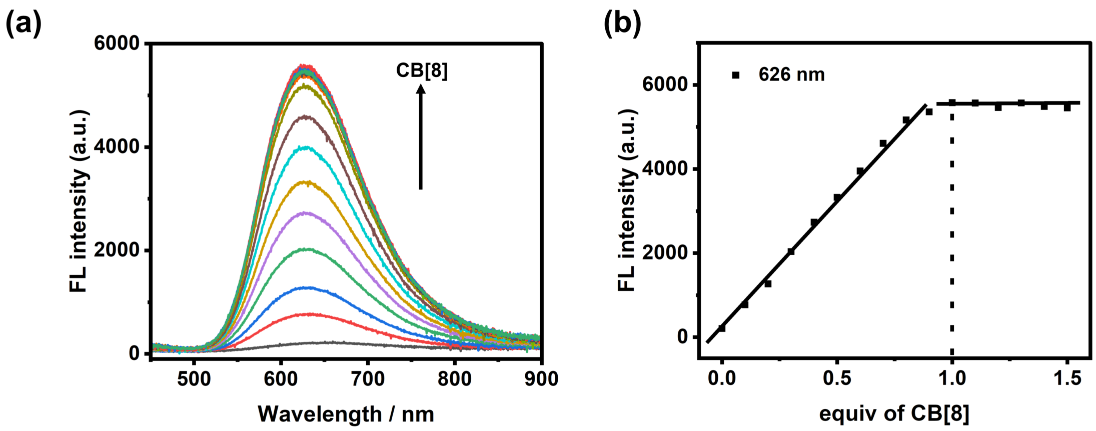

Figure S22. (a) Fluorescence spectra of TPEV (2×10^{-5} M^{-1}) recorded in water upon the addition of CB[8] (λ_{ex} = 420 nm). (b) Plots of the fluorescence maximum versus the equiv. of CB[8].

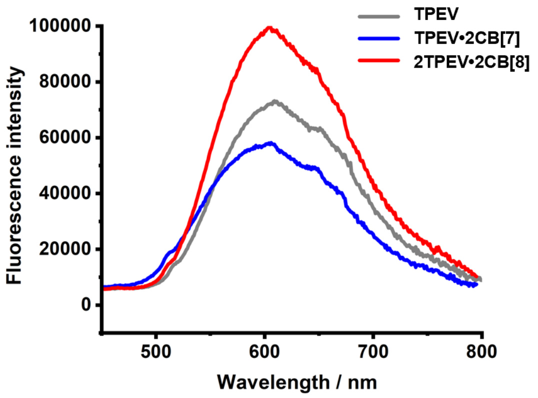

Figure S23. Solid-state fluorescence spectra of TPEV, TPEV·2CB[7], and 2TPEV·2CB[8].

We measured the solid-state fluorescence spectra of all three systems (Figure S23). As expected, all of them show strong emission due to the AIE effect. Notably, 2TPEV·2CB[8] remains the most emissive in the solid state, consistent with its behavior in solution. This highlights that its emission originates from supramolecular dimerization at the molecular level, rather than being state-dependent. Interestingly, we also observed that TPEV·2CB[7] is the least emissive in the solid state, even weaker than free TPEV, which is opposite to the trend seen in solution. We speculate that in solution, CB[7] partially restricts the intramolecular motions of TPEV, leading to a modest fluorescence enhancement. In the solid state, however, free TPEV can

aggregate more effectively, while the bulky CB[7] units in TPEV·2CB[7] may hinder such aggregation, resulting in reduced fluorescence.

References

[S1] (a) Kim, J.; Jung, I.-S.; Kim, S.-Y.; Lee, E.; Kang, J.-K.; Sakamoto, S.; Yamaguchi, K.; Kim, K. *J. Am. Chem. Soc.* **2000**, *122*, 540-541. (b) Day, A.; Arnold, A. P.; Blanch, R. J.; Snushall, B. *J. Org. Chem.* **2001**, *66*, 8094–8100.

[S2] Zhao, S. S.; Wang, L.; Liu, Y.; Chen, L.; Xie, Z., *Inorg. Chem.* **2017**, *56*, 13975-13981.

[S3] Wu, G.; Olesińska, M.; Wu, Y.; Matak-Vinkovic, D.; Scherman, O. A., *J. Am. Chem. Soc.* **2017**, *139*, 3202-3208.
